# ePeak: from replicated chromatin profiling data to epigenomic dynamics

**DOI:** 10.1093/nargab/lqac041

**Published:** 2022-05-27

**Authors:** Maëlle Daunesse, Rachel Legendre, Hugo Varet, Adrien Pain, Claudia Chica

**Affiliations:** Bioinformatics and Biostatistics Hub, Institut Pasteur, Université de Paris, Paris F-75015, France; Flicek Research Group, European Bioinformatics Institute, Cambridge CB10 1SD, UK; Bioinformatics and Biostatistics Hub, Institut Pasteur, Université de Paris, Paris F-75015, France; Plate-forme Technologique Biomics, Centre de Ressources et Recherches Technologiques (C2RT), Institut Pasteur, Université de Paris, Paris F-75015, France; Bioinformatics and Biostatistics Hub, Institut Pasteur, Université de Paris, Paris F-75015, France; Plate-forme Technologique Biomics, Centre de Ressources et Recherches Technologiques (C2RT), Institut Pasteur, Université de Paris, Paris F-75015, France; Bioinformatics and Biostatistics Hub, Institut Pasteur, Université de Paris, Paris F-75015, France; Bioinformatics and Biostatistics Hub, Institut Pasteur, Université de Paris, Paris F-75015, France

## Abstract

We present ePeak, a Snakemake-based pipeline for the identification and quantification of reproducible peaks from raw ChIP-seq, CUT&RUN and CUT&Tag epigenomic profiling techniques. It also includes a statistical module to perform tailored differential marking and binding analysis with state of the art methods. ePeak streamlines critical steps like the quality assessment of the immunoprecipitation, spike-in calibration and the selection of reproducible peaks between replicates for both narrow and broad peaks. It generates complete reports for data quality control assessment and optimal interpretation of the results. We advocate for a differential analysis that accounts for the biological dynamics of each chromatin factor. Thus, ePeak provides linear and nonlinear methods for normalisation as well as conservative and stringent models for variance estimation and significance testing of the observed marking/binding differences. Using a published ChIP-seq dataset, we show that distinct populations of differentially marked/bound peaks can be identified. We study their dynamics in terms of read coverage and summit position, as well as the expression of the neighbouring genes. We propose that ePeak can be used to measure the richness of the epigenomic landscape underlying a biological process by identifying diverse regulatory regimes.

## INTRODUCTION

High throughput techniques aimed at profiling DNA binding proteins and histone modifications genome wide have experienced and accelerated evolution in the past years. After more than 10 years of Chromatin ImmunoPrecipitation with sequencing (ChIP-seq) hegemony (1,2), enzyme-tethering methods such as CUT&RUN and CUT&Tag have been developed to improve the resolution of epigenomic profiles of samples with as few as 100–1000 cells (3,4).

Best practices for ChIP-seq analysis have been defined and validated for a long time by the ENCODE consortium (5,6). Nevertheless, among the many available tools for ChIP-seq analysis most do not include key data processing steps and statistical controls necessary for the robust and reproducible description of the local epigenomic landscape. Such is the case of the metrics to assess the quality of the immunoprecipitation and the Irreproducible Discovery Rate (IDR) (5,7) for the estimation of replicate reproducibility, whose automation has proven to be difficult. For CUT&RUN and CUT&Tag protocols, general pipelines are being developed that incorporate essential adjustments like the spike-in calibration and different peak calling strategies (8,9).

Standards for the differential analysis of RNA-seq datasets have been widely studied and implemented allowing users to do an informed choice of the methods to optimise sensitivity and/or specificity (10,11). This is not yet the case for ChIP-seq, CUT&RUN and CUT&Tag, where most researchers are not aware of the effect that a statistical setting can have on their comparisons, depending on the dynamics of the chromatin factor under scrutiny.

We present ePeak, a pipeline that streamlines the complete analysis process from raw ChIP-seq, CUT&RUN and CUT&Tag data to peak calling with the identification of reproducible peaks if replicates are available. ePeak is compatible with any chromatin factor, e.g. histone modification or transcription factor (TF), and can process at once multiple chromatin factors with different number of biological replicates measured in one or more biological conditions. It is designed to facilitate the analysis when the optimal parameters for each tool are known. However, it also permits to efficiently estimate the optimal parameter values by iterating the analysis without repeating time consuming steps such as mapping and deduplication.

Following peak calling, ePeak also offers a downstream analysis module to evaluate the differential marking or binding between multiple biological conditions. This implies an additional level of complexity that is unknown for RNA-seq experiments. Epigenomic profiling is performed for histone modifications with different nucleation, spreading and maintenance kinetics, as well as proteins with variable affinity to tightly or loosely compacted chromatin. As a consequence, the proper quantification of the marking/binding dynamics needs to consider these differences. ePeak provides a flexible set of statistical settings with multiple options to adjust the quantification and subsequent comparison. These include: linear, nonlinear and spike-in methods for the library size normalisation as well as two different variance estimation approaches, limma (12) and DESeq2 (13).

As an example, we apply the various statistical settings proposed by ePeak on a published dataset (14) of three histone modifications (H3K4me3, H3K27ac and H3K4me1) and two transcription factors (Oct4 and Klf4) profiled with ChIP-seq. We identify the differentially marked/bound peaks between two biological conditions (shControl and shUbc9) for each statistical setting. We show that the choice of normalisation method and variance estimation model results in distinct sets of differentially marked/bound peaks, depending of the chromatin factor. Furthermore we propose that such dependency can be described in terms of the two sources of ChIP-seq variability: one related to the read coverage and the other connected to the position of maximum enrichment or summit. Most importantly, genes neighbouring these differentially marked/bound peak populations show different mean expression changes, which suggests variable transcriptomic regimes and thus potentially diverse regulatory mechanisms.

## MATERIALS AND METHODS

### Workflow description

**Figure 1.**
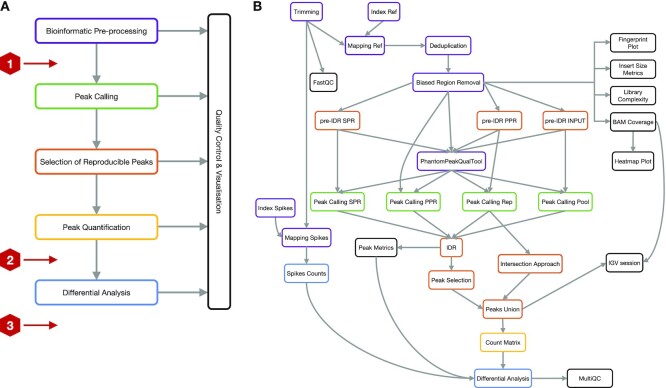
The ePeak workflow. (**A**) Five ePeak modules executing specific and interdependent tasks. Stop signs indicate where the analysis ends, depending on the data provided by the user. Stop 1 if no replicates are available. Stop 2 for datasets with replicates for only one condition. Stop 3 when replicates are available for two or more conditions. (**B**) ePeak Snakemake rule graph illustrating the input/output dependencies between steps. Border colour indicates the module membership of each rule (SPR: self pseudo-replicate, PPR: pooled pseudo-replicate).

ePeak is a modular pipeline designed to deal with various chromatin factors, e.g. histone modifications, transcription factors (TFs), profiled in multiple biological conditions with/without replicates. It is built in five modules that execute specific and interdependent tasks as illustrated in Figure 1A (Supplementary material for detailed description).

The *bioinformatic pre-processing* module aims at obtaining the deduplicated aligned reads. It includes: filtering, trimming and quality assessment of the raw data; mapping to the reference genome, correction of PCR amplification biases and, when available, removal of biased regions (i.e. blacklisted regions) that display artificially high coverage. For experiments where global changes of histone modifications is supposed to take place a spike-in control is added (15), this module executes the same steps for the exogenous chromatin on the corresponding genome.

Next, the *peak calling* and *selection of reproducible peaks* modules are two intermingled routines that provide the ranked list of reproducible narrow or broad peaks. These are identified using the irreproducible discovery rate (IDR) for narrow peaks or the intersection approach for broad peaks (Supplementary Figure S1).

The *peak quantification* module produces the input for the differential analysis, namely the count matrix containing the marking/binding quantification (i.e. the read coverage) of each sample over every peak. Rows of this count matrix correspond to the non-redundant set of reproducible peaks merged among all conditions. This guarantees that the quantitative comparisons are performed between genomic regions of equal length.

Finally, the *differential analysis* module is designed to estimate the significance of the observed changes in marking/binding between the user-defined conditions for each peak in the non-redundant set of reproducible peaks. This module is intended to provide a wide range of statistical settings to obtain a comprehensive quantitative description of the epigenomic-mediated process. Given a proper experimental design, without confounding factors, this module permits to correct unwanted technical variability (i.e. batch effects) and to account for systematic technical variability such as different library sizes. It includes linear and nonlinear methods for normalisation and different variance estimation models as implemented in the DESeq2 and limma packages. For experiments with spike-in control this module performs a linear normalisation using the scaling factor calculated from the mapping coverage of the exogenous chromatin (Supplementary Figure S2).

### ePeak different running modes

ePeak proposes two main running modes, depending on the specific needs of the user: (i) a production mode for users who want to analyse their datasets using standard, predefined parameters for the peak calling (narrow or broad peaks) and for the differential analysis (library size normalisation method and variance estimation approach) and (ii) an exploratory mode for users dealing with epigenomic projects where the behaviour of the chromatin factor under study is uncertain.

Depending on the specific question or the available data, ePeak allows for an analysis at different levels, as illustrated in Figure 1A. A basic analysis stops at the peak calling step when replicates could not be obtained. The outcome in this case will be a list of peaks that can be ranked according to the adjusted *P*-value or the log fold change of the observed enrichment. Else, if biological replicates are available, the pipeline assesses the concordance of peak calls between replicates using the IDR or the intersection procedures, and outputs a list of reproducible peaks. Finally, for complete experimental designs with multiple replicated biological conditions, ePeak performs the differential analysis as defined by the user.

The exploratory mode of ePeak allows to test multiple combinations of peak calling and differential analysis parameters avoiding the re-calculation of intermediate steps. The user can therefore gain a complete understanding of the system while minimising the computing time. Some practical examples of the exploratory mode are: calling narrow and broad peaks on the same pre-processed mapping files; comparing normalisation methods for the differential analysis on the same count matrix.

## RESULTS AND DISCUSSION

### The importance of providing multiple differential analysis settings

Unlike RNA-seq reads that measure the transcriptional output of a given locus, ChIP-seq, CUT&RUN and CUT&Tag reads represent both the probability of marking/binding and its spatial distribution at a given genomic region, i.e. the peak. It follows that these samples have two sources of variability: one related to the read coverage of the peak, and the other to the position of the maximum enrichment or summit. Despite this additional layer of complexity, the differential analysis of marking/binding is traditionally performed using the methods developed for RNA-seq like DESeq2 and limma.

To illustrate the above, we used ePeak to re-analyse a published dataset of three histone modifications (H3K4me3, H3K27ac and H3K4me1) and two TFs (Oct4 and Klf4) profiled using ChIP-seq in two biological conditions (shControl and shUbc9-treated reprogrammable mouse embryonic fibroblasts) (14). We explored the interplay between the above mentioned sources of peak variability and the discriminative power of various statistical settings commonly used for the differential analysis.

**Figure 2.**
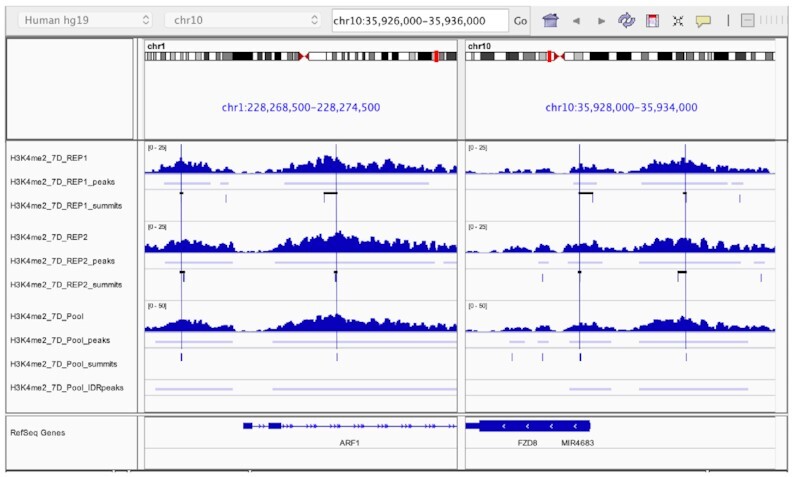
ChIP-seq position variability. Summit instability is defined as the distance between the summit of the peak called in each replicate and the corresponding reproducible peak. Tracks show the IP coverage, peak and summit position for the two replicates separately (top) and pooled (bottom) in two genomic regions. Vertical blue lines indicate the position of the reproducible peak summit and horizontal black lines the distance to the corresponding summit in each replicate.

**Figure 3.**
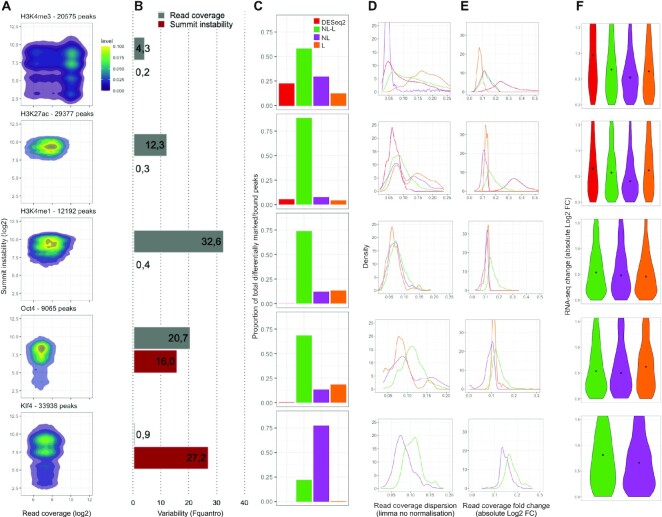
Comparison of the statistical settings for differential analysis. (**A**) Kernel density estimation of read coverage and summit instability for all reproducible peaks across replicates of the two biological conditions under study. (**B**) Read coverage and summit position variability estimation using the Fquantro statistic (16). (**C**) Proportion of total differentially marked/bound peaks obtained using each statistical setting. DESeq2 = DESeq2 with geometric mean normalisation; NL-L = limma with nonlinear and with linear normalisation; NL = limma with nonlinear normalisation only; L = limma with linear normalisation only. (**D, E**) Quantitative characterisation of differentially marked/bound peak populations obtained using each statistical setting. Distribution of ChIP-seq read counts dispersion as estimated by limma (D). Distribution of ChIP-seq absolute changes in read counts between shUbc9 and shControl (E). Colours correspond to panel C. (**F**) Expression dynamics of genes neighbouring differentially marked/bound peak populations. Distribution absolute changes in RNA-seq read counts between shUbc9 and shControl. Colours correspond to panel (C).

For each reproducible peak, we quantified: (i) number of reads per replicate overlapping the peak coordinates, i.e. the read coverage; (ii) the distance between the summit position estimated by pooling the IP replicates and the summit position of the overlapping peak found when using each IP replicate separately, i.e. the summit instability illustrated in (Figure 2). The resulting distribution of read coverage and summit instability for the five chromatin factors is plotted in Figure 3A.

We then estimated the variability of read coverage and summit instability for each chromatin factor, using the Fquantro statistic (Figure 3B), a ratio of the mean squared error between-conditions and the mean squared error within-conditions (16). Distinct patterns of read coverage and/or summit instability variability between shControl and shUbc9 conditions can be observed across the five chromatin factors (Figure 3A and B). We thus investigated how such patters can influence the differential analysis results for each modification/TF. We determined the proportion of differentially marked/bound peaks identified using the statistical settings available in ePeak: DESeq2 with linear normalisation (geometric mean); limma with linear (scalar) or nonlinear normalisation (quantile or cyclic loess) (Figure 3 C).

Depending on the modification/TF, DESeq2 permits the identification of 3–25% of the total differentially marked/bound peaks, which are also found by limma independently from the normalisation method. The size of this set of differentially marked/bound peaks diminishes as the read coverage and summit position variability between conditions increases for the different modifications/TFs (Figure 3B and C). This result is compatible with the fact that those peaks have the lowest read coverage dispersion (i.e. variability between replicates) as estimated by limma (Figure 3D). This is also in agreement with the observation that, for RNA-seq differential analysis, the count based model used in DESeq2 for dispersion estimation is a more conservative approach than the linear models used in limma for variance estimation (17,18).

For the results obtained using limma, three main classes of modifications/TFs can be described (Figure 3B and C):

- Stable modification (H3K4me3) with low variability of read coverage and summit position between conditions. Significant marking differences depend on the statistical setting, in particular on whether a linear or nonlinear normalisation method is used.
- Modifications (H3K27ac and H3K4me1) with increased read coverage variability between conditions and TF (Oct4) with medium variability for both read coverage and summit position. Differential analysis results are mostly independent from the statistical setting but an additional set of differentially marked/bound peaks (12–35% of the total) can be obtained by using specifically a linear or nonlinear normalisation method.
- TF (Klf4) with high summit position variability and no read coverage variability. More than 75% of the significant differences are detected with a single statistical setting, in this case limma with nonlinear normalisation.

Not surprisingly, there is no univocal relationship between the number of differentially marked/bound peaks and their quantitative characteristics like the read coverage dispersion or fold change between conditions (Figure 3D and E). The largest population of differentially marked/bound peaks does not always show the lowest dispersion. Moreover, depending on the modification/TF, a given statistical setting like limma with linear normalisation can pinpoint significant differences among peak sets with low (H3K4me1 or Oct4) or high (H3K4me3) dispersion (Figure 3D). The sole exception is the peak population identified by the DESeq2 approach that, as mentioned above, always contains peaks with lowest read coverage dispersion (Figure 3D).

We finally tested the expression distribution of the genes associated to the differentially marked/bound peak populations described above. We selected the genes located within 5K or 30K from those peaks and plotted their absolute RNA-seq log fold change between shControl and shUbc9 (Figure 3F). Each peak population is linked to genes with distinct expression changes, thus highlighting the biological interest of considering the whole range of differential marking/binding results when studying epigenomic dynamics.

Even if the trends we describe can vary depending on the biological system under study, our observations show that multiple statistical settings for the differential analysis of marking/bind-ing can provide a quantitative description of the various changes that a chromatin factor may undergo, and ultimately inform us about its regulation mode.

### ePeak in comparison to available tools

Since the first publication of the ChIP-seq protocol (1,2) a plethora of methods have been developed and implemented in order to: (i) identify genomic regions with a significant enrichment for the profiled chromatin factor, i.e. peak calling and (ii) compare the enrichment between biological conditions, i.e. differential analysis.

There is one broad category of methods dedicated to the differential analysis step: DiffBind (19), ChIPQC (20), ChIPComp (21), DBChip (22), MMDiff (23), MAnorm (24). They mostly differ on the normalisation strategy used to account for systematic technical variability and the statistical test chosen to evaluate the significance of the difference in enrichment per region. Another category of methods perform peak calling and differential analysis: SICER2 (25), MACS2 (26), HOMER (27), RSEG (28), but only diffReps (29), MultiGPS (30) and PePr (31) can take into account replicates for the differential analysis (Supplementary Table S1).

More recently, ChIP-seq pipelines that take care of the intermediate steps from the raw data to the peak calling have been implemented. The GenePipes pipeline (32) includes the calculation of quality control metrics like cross-correlation and sequence bias as well as the functional characterisation of peaks according to their genomic localisation or enriched sequence motifs. However it does not cover the differential analysis. The snakePipes ChIP-seq pipeline (33) does not perform any quality control step but does the differential analysis with the R package CSAW. The nf-core (nextflow) pipeline (34) comprises both quality control metrics calculation and differential analysis using the R package DESeq2 and can be applied on narrow and broad peaks.

Regarding CUT&RUN and CUT&Tag, the nf-core (nextflow) pipeline (8) and the CUT&RUNTools 2.0 (9) are currently the most complete protocols. None of them, however, incorporate the differential marking/binding step.

To our knowledge, there is no current pipeline that, like ePeak, can perform all the following tasks for ChIP-seq, CUT&RUN and CUT&Tag datasets: (i) peak calling starting from the raw sequencing data for both narrow and broad peaks (ii) identification of reproducible peaks between replicates for both narrow and broad peaks, using respectively the Irreproducible Discovery Rate (IDR) and the intersection approach (iii) differential analysis with multiple statistical settings.

Last but not least, ePeak produces (i) a customised MultiQC with quality report of all the pre-processing steps for all datasets (Supplementary Figure S3), (ii) an XML session readable by the Integrative Genomic Viewer (IGV) and (iii) a complete report with the results of the differential analysis.

### Beyond an analysis pipeline

On top of being a complete and general ChIP-seq, CUT&RUN and CUT&Tag analysis pipeline, ePeak is a flexible tool to systematically explore the epigenomic landscape underlying a dynamic biological system. It permits the exhaustive exploration of the marking/binding changes that follow or drive the perturbation (e.g. mutation, treatment, pathogen infection) of a steady or control state.

Indeed, the various statistical settings of the differential analysis module enable a quantitative estimation of the heterogeneity of the regulatory response. The distinct populations of differentially marked/bound peaks can be interpreted as groups of genomic regions with differing regulatory roles, e.g. related to leaky or not synchronised transcriptional events, or to changes occurring only in a cell sub-population.

Our results on the statistical settings used for the differential analysis of three histone modifications and two TF exemplify the complex relation between the sources of ChIP-seq variation and the ability to identify significant marking/binding changes. Assuming that unwanted technical variation is corrected or accounted for, epigenomic profiling techniques are measuring a dynamic behaviour resulting from the interplay between the intrinsic regulatory characteristics of a chromatin factor and the genomic/nuclear/cellular environment shaped by the biological process under study. These intrinsic factors may include, among others, the writing/reading/erasing kinetics of the histone modifications or the binding affinity of the TFs and their ability to target compacted chromatin. While the sole epigenomic profiling is not enough to deconvolute all those intrinsic and environmental aspects, it can give a sense of the variety of regulatory possibilities. Considering all the above, ePeak can be used to measure the richness of the epigenomic landscape and identify potentially diverse regulatory regimes.

## DATA AVAILABILITY

The pipeline is freely available at https://gitlab.pasteur.fr/hub/ePeak/ under GNU General Public Licence. All needed tools are packaged under a singularity container published on the Cloud Library. All the data used in this study can be accessed at the Gene Expression Omnibus resource under the GEO accession GSE99009.

## Supplementary Material

lqac041_Supplemental_Files
