# Supplementary material for: ePeak: from replicated chromatin profiling data to epigenomic dynamics
Source: NAR Genom Bioinform. 2022 May 27;4(2):lqac041. doi: 10.1093/nargab/lqac041 (PMC9154330; doi:10.1093/nargab/lqac041)
Supplement: lqac041_Supplemental_Files [file lqac041_supplemental_files.zip › ePeak_NARGAB_SupplementaryMethods.pdf]

ePeak: from replicated chromatin profiling data to epigenomic dynamics

Maëlle Daunesse, Rachel Legendre, Hugo Varet, Adrien Pain, Claudia Chica

Extended methods **1**
Bioinformatic pre-processing module 2
Peak calling module 3
Selection of reproducible peaks 3
Differential analysis 5
Analysis report 6

Extended methods

ePeak includes a combination of Snakemake [1] and python code. It follows the good practices of reproducibility described by the Snakemake's authors. The workflow is stored in a dedicated git repository:
`https://gitlab.pasteur.fr/hub/ePeak`.

The pipeline is stored in the Snakefile, and all the rules are stored in the workflow/ directory. One rule corresponds to one software/step and we can split the pipeline into five modules (Figure 1A main text):

- Bioinformatic pre-processing: all samples are treated independently according to the experimental design.
- Peak calling: IP and INPUT are matched for each modification/TF and condition.
- Selection of reproducible peaks: IPs are evaluated by replicates, self pseudo-replicates and pooled pseudo-replicates for each modification/TF and condition.
- Peak quantification: reproducible peaks of all conditions are merged for each modification/TF and read counts are computed per IP sample.
- Differential analysis: performed per modification/TF.

The user fills a YAML file to configure each step of the pipeline (configuration file) and a tabulated file that summarise the biological design (design file). The pipeline starts by reading the configuration and design files. Then, it checks the correspondence between the information in the configuration file, the design file and the raw data file names. In case of discordant information or files missing, the pipeline does not start. Finally, the design file is analysed to determine which module(s) can be run and where the pipeline will end.

For example, if the user chooses to perform the differential analysis but replicates are not provided, the pipeline will automatically stop after the peak calling step. Seemingly, a minimum of two replicates by IP and one INPUT are required to obtain reproducible peaks; at least 2 conditions per chromatin factor and reproducible peaks are required to perform the differential analysis (Figure 1A main text). Since ePeak is processing each chromatin factor independently, it can run until the reproducible peaks selection for one dataset, and still run the differential analysis module for another one.

Bioinformatic pre-processing module

The bioinformatic pre-processing module includes quality control of reads, adapters' trimming, mapping, read deduplication, removal of biased regions, if possible, and calculation of quality metrics. Some of these rules were adapted from the RNA-seq pipeline of Sequana [2].

The tools used within this module are (Figure 1B main text):

- FastQC for quality control of samples [3].
- Cutadapt for filtering reads and trimming adapters [4].
- bowtie2 for read mapping [5]. Mapping is performed against the IP reference genome, and is also done against the control reference genome for spike-in samples. For user-provided genome sequences, a dedicated rule handles the genome indexing.
- MarkDuplicates from the Picard tools suite for removal of duplicated reads [6]. This step can be tailored to keep a specific number of duplicated reads per position, a necessary condition to achieve enough signal for the peak calling in small bacterial genomes [7].
- intersectBed from the bedtools suite [8] for removal of biased regions. This rule is optional, because biased regions have not been identified for many non-model organisms.

This module includes quality assessment procedures specific to ChIP-seq, CUT&RUN and CUT&Tag. It offers multiple tools, which are key to identify the technical problems that can explain poor peak calling or reproducibility results:

- EstimateLibraryComplexity from the Picard tools suite, to evaluate the approximate number of unique molecules sequenced per sample [6].
- CollectInsertSizeMetrics from the Picard tools suite, to calculate the insert size distribution [6].
- plotFingerprint from the deepTools python tools suite, to compare the signal of the IP and INPUT samples, by comparing their cumulative distribution of genome coverage [9].

- PhantomPeakQualTools for the calculation of cross-correlation metrics useful for ChIP-seq experiments [10]. The R script is embedded within ePeak and is stored in the workflow/scripts/ directory.
- plotHeatmap to calculate the gene body coverage profile of IP samples, from the deepTools python tools suite[9].

For samples without replicates, the pipeline stops at this point (Figure 1A main text).

Peak calling module

After the pre-processing module, enriched regions can be identified with the MACS2 and SEACR peak callers, depending on the technique used in the epigenomic profiling [11, 12].

MACS2 can be run using either narrow or broad mode depending on the expected size of the binding site or the histone marking pattern. Two inputs are required for the *macs2* rule: a BAM file corresponding to the IP and one corresponding to the INPUT. IP and INPUT BAM files are paired for each chromatin factor and condition, according to the design file. Peaks can only be called on an IP with a matched INPUT. If several INPUT BAM files are provided corresponding to different replicates, the first one is used for all IP replicates. This approach assumes that there is no significant variability between INPUT replicates.

In addition, there is a setting (see *no_model* parameter) in the configuration files that allows the use of PhantomPeakQualTools's fragment size estimation instead of MACS2' one. This is particularly useful when MACS2 fails to calculate the fragment size using the default model approach.

For data with low read depth of both IP and INPUT samples such as CUT&RUN and CUT&Tag, the SEACR peak caller is available. Bedgraph files are calculated for each sample from the mapped and deduplicated reads. Coverage can be normalised using the spike-in factor calculated from the control genome, a calibration step recommended for these techniques [13]. Then, the IP and corresponding INPUT bedgraph files are provided to SEACR to call peaks using either the stringent or relaxed mode.

Finally, output files are stored in a directory with explicit names specifying the peak caller (MAC2, SEACR), peak calling mode (narrow or broad for MACS2 or stringent and relaxed for SEACR) and the fragment size estimation method (model or no model for MACS2). This allows the user to run this module several times with different parameters and avoid over-writing the results.

Selection of reproducible peaks

The next step is to select the reproducible peaks between biological replicates. The protocol for this depends on the peak calling mode. As a default, the Irreproducible Discovery Rate (IDR) procedure is used for narrow peaks and an in-house optimised intersection approach for broad peaks.

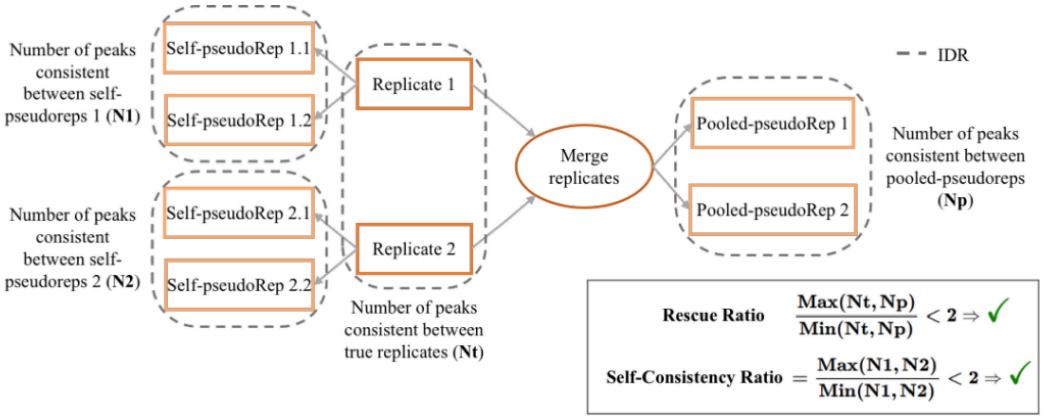

Figure 1: Irreproducible Discovery Rate (IDR) procedure as described in [10]. Steps of the IDR are represented inside dashed line boxes. IDR metrics used for the assessment of the replicates' reproducibility are shown inside the continuous line box.

The automated IDR procedure of ePeak corresponds to the one described by [10]. It starts with a pre-computing step (Figure 1B main text) and provides a set of IDR metrics (Figure 1) that are used for the assessment of the replicates' reproducibility and the final selection of reproducible peaks.

To compute the IDR per peak multiple files have to be pre-computed. Those files are produced by the pre-IDR submodule as defined below:

- *preIDR_SPR* rule generates two IP self pseudo-replicates for each IP replicate by randomly splitting the mapped reads into two BAM files.
- *preIDR_PPR* rule generates two IP pooled pseudo-replicates by merging all mapped IP reads in a single BAM and then randomly splitting it into two BAM files.
- *preIDR_Pool* rule generates, if INPUT replicates are provided, a pooled INPUT BAM file.

This step takes place before the peak calling. Peaks are called on those seven BAM files, with the INPUT as control except for the pooled IP for which the pooled INPUT is used as control if available.

Then the IDR score is estimated for each peak of the IP replicates, self pseudo-replicates and pooled pseudo-replicates. The rescue ratio and self-consistency ratio are calculated as described by [10]. Finally, the reproducible peaks are selected among the peaks called on the pooled IPs and having the lowest IDR score.

The IDR procedure is optimised for narrow peaks. For this reason we included the intersection approach to choose reproducible broad peaks. First, peaks are filtered by selecting those with a q-value lower than 0.01 and a log fold change higher than 3. Second, the proportion of overlap per peak between replicates is determined using intersectBed (from BEDtools suite). Only peaks with an overlap of at least 80% of the longest peak length are kept. The user can force the use of the intersection approach for narrow peaks.

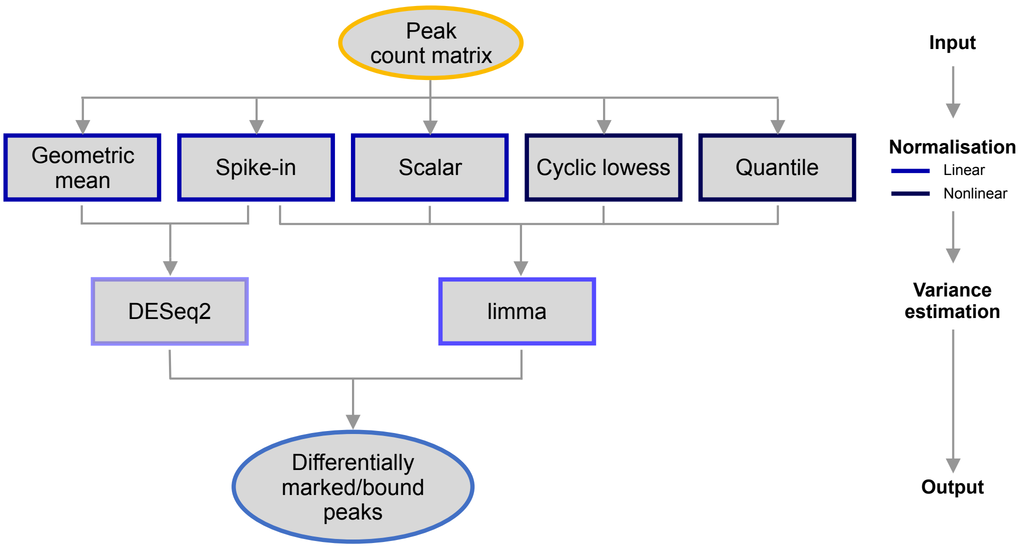

Figure 2: ePeak differential analysis module with the statistical settings implemented in ChIPflowR. Possible combinations of normalisation method and variance estimation approach are shown.

To select reproducible peaks from SEACR only the intersection approach is available, since the IDR has not yet been calibrated for CUT&RUN and CUT&Tag data. Indeed, SEACR is optimised to minimize the number of false positives and tends to produce few peaks, whereas the IDR requires a sufficiently large number of peaks from both signal and noise distributions to identify the reproducible ones.

Differential analysis

Differential analysis is performed for modifications/TFs matching the following requirements:

- A minimum of two biological conditions.
- Two replicates for each condition from which reproducible peaks have been selected.

A union peak file is obtained by merging the reproducible peaks coordinates for all the conditions of one modification using mergeBed from BEDtools with default parameters. Merged peak summit is calculated as the mean of all merged peak summit positions.

The count matrix is then generated for all IP BAM files using featureCounts [14] and the peak coordinates in the union peak file.

The differential analysis is performed to detect differentially marked/bound peaks between the biological conditions. For this, we adapted the SARTools R package [15] and created ChIPflowR which is freely available at `https://gitlab.pasteur.fr/hub/ChIPflowR`. Two approaches to model variability (DESeq2 and limma) as well as linear and nonlinear normalisation methods (scalar, quantile, cyclic loess, spike-in) are

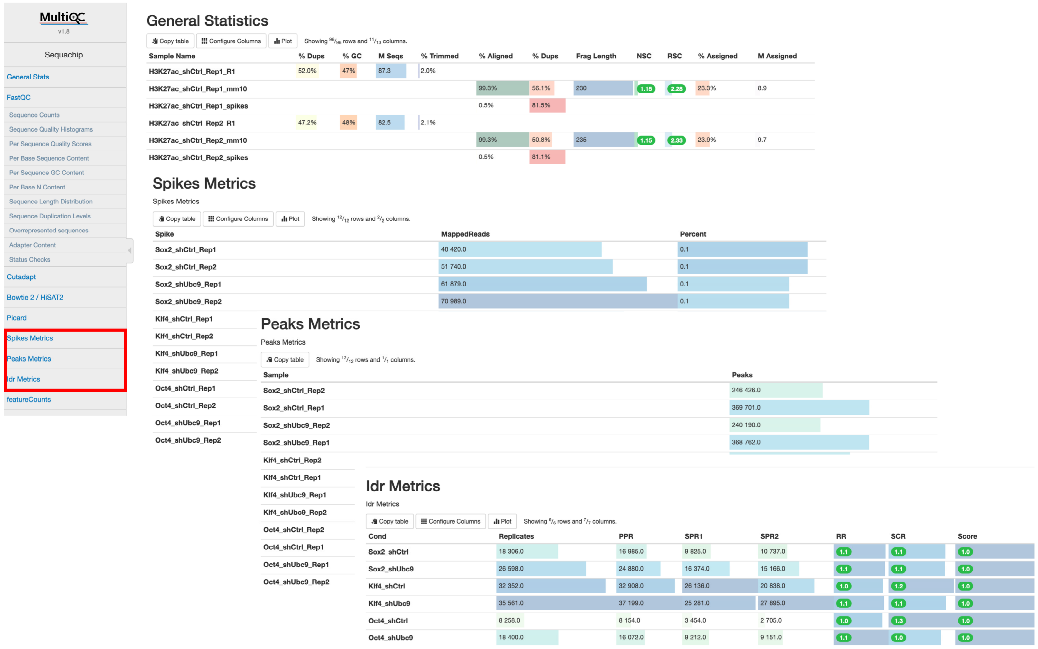

Figure 3: Customised MultiQC report. All classical MultiQC modules are included to provide an interactive report of every step in the analysis. Modules that were added to adapt the report for ChIP-seq, CUT&RUN and CUT&Tag datasets are highlighted in red and presented as partial screenshots.

available to perform the differential analysis (Figure 2). Using the count matrix generated by featureCounts, ChIPflowR carries out the quality assessment of the data using dedicated plots, runs the differential analysis and exports (i) a Rmarkdown HTML report describing the analysis process and (ii) the tables containing the differentially marked/bound peaks coordinates and corresponding statistics.

Analysis report

Finally, a MultiQC [16] report is compiled, which concatenates all relevant information and metrics from the log files produced during the pipeline execution. It provides a global view of the results (Figure 3).

A particular effort was put in the customisation of the MultiQC config file aiming at offering a complete and interactive final report. In addition to the classic MultiQC modules such as bowtie2 or picard-tools, we added homemade chunks: a table summarising the number of peaks called per IP dataset; for spike-in datasets, a table containing the mapping statistics on the exogenous genome. Moreover, a whole section is dedicated to the IDR results. Rescue Ratio, Self-Consistency Ratio and IDR score are tabulated for replicates, self pseudo-replicates and pooled pseudo-replicates, facilitating the identification of reproducible replicates. To further simplify the quality assessment, samples that failed or succeeded according to the PhantomPeakQual tools and the IDR metrics are highlighted using a MultiQC formatting rule.

ePeaks also produces an XLM session readable by the Integrative Genomic Viewer

(IGV) [17]. It includes the coverage tracks (bigwig format) and peaks (bed format) for all the IP samples in the design, coloured by mark/TF. Users can further modify this session according to their visualisation needs.

References

[1] J. Koster and S. Rahmann. Snakemake–a scalable bioinformatics workflow engine. *Bioinformatics*, 28(19):2520–2522, October 2012.

[2] Thomas Cokelaer, Dimitri Desvillechabrol, Rachel Legendre, and Mélissa Cardon. 'sequana': a set of snakemake ngs pipelines. *Journal of Open Source Software*, 2(16):352, 2017.

[3] Simon Andrews, Felix Krueger, Anne Segonds-Pichon, Laura Biggins, Christel Krueger, and Steven Wingett. FastQC. Babraham Institute, January 2012.

[4] Marcel Martin. Cutadapt removes adapter sequences from high-throughput sequencing reads. *EMBnet.journal*, 17(1):10–12, 2011.

[5] B. Langmead and S. L. Salzberg. Fast gapped-read alignment with Bowtie 2. *Nat Methods*, 9(4):357–359, Mar 2012.

[6] Picard toolkit. `http://broadinstitute.github.io/picard/`, 2019.

[7] Maria-Vittoria Mazzuoli, Maëlle Daunesse, Hugo Varet, Isabelle Rosinski-Chupin, Rachel Legendre, Odile Sismeiro, Myriam Gominet, Pierre Alexandre Kaminski, Philippe Glaser, Claudia Chica, Patrick Trieu-Cuot, and Arnaud Firon. The CovR regulatory network drives the evolution of Group B Streptococcus virulence. *PLOS Genetics*, 17(9):e1009761, September 2021.

[8] A. R. Quinlan and I. M. Hall. BEDTools: a flexible suite of utilities for comparing genomic features. *Bioinformatics*, 26(6):841–842, Mar 2010.

[9] Fidel Ramírez, Devon P Ryan, Björn Grüning, Vivek Bhardwaj, Fabian Kilpert, Andreas S Richter, Steffen Heyne, Friederike Dündar, and Thomas Manke. deepTools2: a next generation web server for deep-sequencing data analysis. *Nucleic Acids Research*, 44(W1):W160–W165, July 2016.

[10] S. G. Landt, G. K. Marinov, A. Kundaje, P. Kheradpour, F. Pauli, S. Batzoglou, B. E. Bernstein, P. Bickel, J. B. Brown, P. Cayting, Y. Chen, G. DeSalvo, C. Epstein, K. I. Fisher-Aylor, G. Euskirchen, M. Gerstein, J. Gertz, A. J. Hartemink, M. M. Hoffman, V. R. Iyer, Y. L. Jung, S. Karmakar, M. Kellis, P. V. Kharchenko, Q. Li, T. Liu, X. S. Liu, L. Ma, A. Milosavljevic, R. M. Myers, P. J. Park, M. J. Pazin, M. D. Perry, D. Raha, T. E. Reddy, J. Rozowsky, N. Shoresh, A. Sidow, M. Slattery, J. A. Stamatoyannopoulos, M. Y. Tolstorukov, K. P. White, S. Xi, P. J. Farnham, J. D. Lieb, B. J. Wold, and M. Snyder. ChIP-seq guidelines and practices of the ENCODE and modENCODE consortia. *Genome Research*, 22(9):1813–1831, September 2012.

[11] Yong Zhang, Tao Liu, Clifford A Meyer, Jérôme Eeckhoute, David S Johnson, Bradley E Bernstein, Chad Nussbaum, Richard M Myers, Myles Brown, Wei Li, and X Shirley Liu. Model-based Analysis of ChIP-Seq (MACS). *Genome Biology*, 9(9):R137, 2008.

[12] Michael P. Meers, Dan Tenenbaum, and Steven Henikoff. Peak calling by Sparse Enrichment Analysis for CUT&RUN chromatin profiling. *Epigenetics & Chromatin*, 12(1):42, December 2019.

[13] Ye Zheng, Ahmad Kami, and Henikoff Steven. Cut&tag data processing and analysis tutorial. `https://dx.doi.org/10.17504/protocols.io.bjk2kkye`, 2020.

[14] Y. Liao, G. K. Smyth, and W. Shi. featureCounts: an efficient general purpose program for assigning sequence reads to genomic features. *Bioinformatics*, 30(7):923–930, Apr 2014.

[15] Hugo Varet, Loraine Brillet-Guéguen, Jean-Yves Coppée, and Marie-Agnès Dillies. SARTools: A DESeq2- and EdgeR-based r pipeline for comprehensive differential analysis of RNA-seq data. *PLOS ONE*, 11(6):e0157022, 2016.

[16] P. Ewels, M. Magnusson, S. Lundin, and M. K?ller. MultiQC: summarize analysis results for multiple tools and samples in a single report. *Bioinformatics*, 32(19):3047–3048, 10 2016.

[17] H. Thorvaldsdottir, J. T. Robinson, and J. P. Mesirov. Integrative Genomics Viewer (IGV): high-performance genomics data visualization and exploration. *Briefings in Bioinformatics*, 14(2):178–192, March 2013.
